# Omega-3 Status and the Relationship between Plasma Asymmetric Dimethylarginine and Risk of Myocardial Infarction in Patients with Suspected Coronary Artery Disease

**DOI:** 10.1155/2012/201742

**Published:** 2012-12-31

**Authors:** Heidi Borgeraas, Elin Strand, Eva Ringdal Pedersen, Jutta Dierkes, Per Magne Ueland, Reinhard Seifert, Eirik Rebnord Wilberg, Pavol Bohov, Rolf K. Berge, Dennis W. T. Nilsen, Ottar Nygård

**Affiliations:** ^1^Institute of Medicine, Haukeland University Hospital, 5021 Bergen, Norway; ^2^Department of Heart Disease, Haukeland University Hospital, 5021 Bergen, Norway; ^3^Division of Cardiology, Stavanger University Hospital, 4011 Stavanger, Norway

## Abstract

*Background*. Asymmetric dimethylarginine (ADMA) is an endogenous inhibitor of nitric oxide synthase. A previous rat study revealed an ADMA lowering effect following treatment with omega-3 polyunsaturated fatty acids (n-3 PUFAs). We sought to examine if an association between plasma ADMA and risk of acute myocardial infarction (AMI) was modified by serum n-3 PUFA status. *Methods*. The cohort included 1364 patients who underwent coronary angiography for suspected coronary artery disease in 2000-2001. Fatal and nonfatal AMI events were registered until December 31, 2006. Risk associations with AMI were estimated across ADMA quartiles (linear trend) and the upper decile. *Results*. No association between concentration of any n-3 PUFA and ADMA was observed. Only ADMA levels in upper decile were significantly associated with AMI with a multivariate adjusted hazard ratio (HR) (95% confidence interval) versus the rest of the population of 2.11 (1.34, 3.32). The association was strengthened among patients with below median levels of **α**-linolenic acid (ALA) (HR 3.12 (1.64, 5.93)), but was only influenced by longer chain n-3 PUFA after additional adjustments for HbA1c, estimated glomerular filtration rate, and hypercholesterolemia. *Conclusions*. The association of ADMA with risk of AMI is influenced by serum n-3 PUFA and particularly ALA.

## 1. Introduction

An early and critical event in the pathogenesis of cardiovascular disease (CVD) is endothelial (vasodilator) dysfunction. Normal endothelial function depends on adequate levels of nitric oxide (NO), which acts as a vasodilator, inhibits the excessive proliferation of vascular smooth muscle cells [1], enhances endothelial cell survival and proliferation [2], and suppresses the adhesion of platelets and inflammatory cells to the vessel wall [3].

NO is synthesized from the amino acid L-arginine by a family of NO synthase enzymes (NOS). Asymmetric dimethylarginine (ADMA) acts as an inhibitor of NOS and thus decreases the synthesis and availability of NO. A high plasma level of ADMA is regarded as an independent predictor of CVD and is also associated with end stage renal disease [4].

Altered activity of the ADMA metabolizing enzymes, dimethylarginine dimethylaminohydrolase I and II (DDAH-I and DDAH-II), has been suggested as a possible cause for plasma ADMA accumulation. DDAH activity is directly downregulated by reactive oxygen species (ROS) generated by high glucose levels [5], oxidized LDL cholesterol (oxLDL), and the cytokine, tumor necrosis factor *α* (TNF-*α*) [6]. Additionally, the expression of endothelial cell protein arginine N-methyltransferases (PRMT), the enzymes which synthesize ADMA, is upregulated in the presence of oxLDL [7].

Studies have revealed altered DDAH activity through activation of peroxisome proliferator-activated receptor *γ* (PPAR*γ*) [8] and sterol regulatory binding protein 1c and 2 (SREBP-1c and SREBP-2) [9]. Activation of PPAR*γ* upregulates DDAH-II expression and enzyme activity [8]. Inhibition of SREBP-1c upregulates DDAH-I expression and activity, while inhibition of SREBP 2 has the opposite effects [9]. Fatty acids (FAs) are natural ligands for PPAR*γ* [10] and SREBPs [11], and omega-3 polyunsaturated FA (n-3 PUFA) may act as PPAR*γ* agonists [10] and SREBP-1c antagonists [11].

n-3 PUFAs include the plant-derived *α*-linolenic acid (ALA) and the fish oil-derived eicosapentaenoic acid (EPA), docosapentaenoic acid (DPA), and docosahexaenoic acid (DHA). Although both groups of n-3 PUFA may have cardiovascular protective properties, the clinical implications of a high intake of n-3 PUFA derived from plant or fish oil in secondary prevention of coronary artery disease (CAD) are still controversial [12–14].

Studies investigating the association between n-3 PUFA and ADMA are scant and inconsistent. A randomized intervention trial, among men with long-standing hyperlipidemia, revealed no differences in ADMA levels after n-3 PUFA supplementation [15]. However, a prospective study revealed lower plasma ADMA concentrations in rats treated with EPA and DHA compared with rats given olive oil [16], and ingestion of a high fat meal, in diabetes patients, has been associated with elevated plasma ADMA levels [17].

The aim of the present study was to investigate if n-3 PUFA influences the association between ADMA levels and risk of AMI in patients with coronary heart disease, hypothesizing that the relationship would be the strongest in patients with impaired n-3 PUFA status.

## 2. Methods

### 2.1. Study Population

The Bergen coronary angiography cohort (BECAC) includes 3718 patients who underwent coronary angiography for suspected CAD during 2000–2004. The majority (92%) had stable angina. The present study included 1364 initial patients recruited to BECAC during 2000-2001. More than half of these patients (*n* = 707) did also participate in the Western Norway B Vitamin Intervention Trial (WENBIT), an RCT that investigated the effect of high dose B vitamin supplementation on risk of CVD and mortality [18]. About 80% of the WENBIT participants completed a semiquantitative food-frequency questionnaire (FFQ) at trial enrolment, providing information on dietary habits during the last year [19]. The study protocol met the mandate of the Declaration of Helsinki and was approved by the Regional Committee for Medical Research Ethics and the Norwegian Data Inspectorate. A signed consent form was obtained from all participants.

### 2.2. Baseline Data

Information about medical history, risk factors and medications were provided through a self-administered questionnaire completed by each patient as previously reported [18]. Hypertension and diabetes mellitus (DM) were classified by preexisting diagnosis, and DM includes both type 1 and 2. Smokers included self-reported current smoking, those who quit smoking within <1 month, and patients with plasma cotinine > 85 ng/mL [20]. Family history of CAD included those reporting to have at least one 1st degree relative suffering from CAD before the age of 55 for men and 65 for women. Information from the questionnaires was checked against medical records. Fasting was referred to as not having ingested any food 6 hours prior to blood sample collection. Untreated serum levels of total cholesterol ≥ 6.5 mmol/L were regarded as hypercholesterolemic. Left ventricular ejection fraction (LVEF) (%) was determined by ventriculography or echocardiography and values < 50% were considered as impaired. The extent of CAD was angiographically verified and scored 0 to 3 according to the number of main vessels with significant diameter stenosis (≥50%).

### 2.3. Endpoint and Followup

The participants were followed from angiography in 2000 or 2001 and until they experienced an acute AMI or throughout December 31, 2006.

Information on clinical events was collected from hospitals and from the Norwegian Cause of Death Registry. AMI definition, published in 2000 [21], was used as diagnostic criteria. Procedure-related nonfatal AMI occurring within 24 h of coronary angiography, percutaneous coronary intervention (PCI), or coronary artery bypass graft surgery (CABG) was excluded from the endpoint. All events were adjudicated by members of the endpoints committee.

### 2.4. Biochemical Analyses

Serum samples were collected before angiography and stored at −80°C until analysis. Serum apolipoprotein A-I, apolipoprotein B, and lipoprotein (a) were measured on the Hitachi 917 system (Roche Diagnostics, GmbH, Mannheim, Germany). C-reactive protein (CRP) was determined using a latex, high sensitive assay (Behring Diagnostics, Marburg, Germany). Serum fatty acid methyl esters were extracted by treatment of serum with 2% (v/v) of sulfuric acid in methanol [22] and analyzed by gas-liquid chromatography (GC 8000 TOP, Finnigan, USA) on DB1-ms capillary column (j & W Scientific, USA) coupled to a flame-ionization detector [23]. Within-day coefficient of variation (CV) was 1.4% for total FAs (TFAs) (mg/L) and 0.37% for ALA (wt%). Within-day CV for the combination of the long chain n-3 PUFA (n-3 LCPUFA) EPA, DPA, and DHA (wt%) was 2.2% and ranged between 0.97% and 1.88% for the individual n-3 LCPUFA. Plasma ADMA was determined by high performance liquid chromatography/tandem mass spectrometry (LC-MS/MS) at BEVITAL AS (http://www.bevital.no/), and within-day CV was 4%. Cotinine was measured by LC-MS/MS [24]. LDL cholesterol was calculated by using the Friedewald formula, and estimated glomerular filtration rate (eGFR) was calculated using the Chronic Kidney Disease Epidemiology Collaboration [25].

### 2.5. Statistical Methods

Continuous variables are presented as means (±SD) and categorical variables as counts (percentage). Mean trends over plasma ADMA quartiles were estimated using linear regression for continuous variables and logistic regression for binary variables.

Hazard ratios of AMI events over quartiles of plasma ADMA and for ADMA as a dichotomous variable (cutoff at 90th percentile) were estimated with Cox proportional hazard models. Nonlinear effects were additionally investigated with GAM plots using penalized smoothing splines for the functional form of the covariate [26]. The adjusted model included age (continuous), sex, acute coronary syndrome (ACS; yes/no), DM (yes/no), hypertension (yes/no), current smoking (yes/no), extent of significant CAD (no significant CAD, 1 vessel disease, 2 vessel disease and 3 vessel disease (0–3)), and LVEF (continuous). HbA1c (continuous), hypercholesterolemia (yes/no), and eGFR (continuous) adjustments were included in an additional model. Effect modifications by serum levels of TFAs, ALA, n-3 LCPUFA, or total n-3 PUFA (ALA plus n-3 LCPUFA) were investigated by including dichotomous transformed cofactors of the respective FA as interaction terms in the Cox model.

All probability values are 2-tailed and were considered significant when <0.05. Statistical analyses were performed with SPSS 18 (SPSS Inc., Chicago, IL, USA) and R 2.14.2 (the R Foundation for Statistical Computing, Vienna, Austria).

## 3. Results

### 3.1. Baseline Characteristics

Baseline characteristics of the 1364 participants, according to quartiles of plasma ADMA concentrations, are presented in Table 1. Mean (±SD) plasma ADMA concentrations were 0.45 (0.05) and 0.82 (0.11) *μ*mol/L for quartile 1 and 4, respectively, and 0.92 (0.11) for the upper decile. The overall mean age was 61.0 years and 74.7% were men. Higher ADMA levels were associated with increasing age and higher proportion of female gender. BMI showed a negative association with ADMA quartiles, which, however, disappeared after adjustment for age and sex (data not shown). There was no association between fasting status and ADMA after adjustment for age and sex (data not shown). FFQ data on dietary habits during last year were available from 705 patients who also participated in WENBIT, and the mean (SD) intake of fish was 119 (67.7) g/d and 116 (63.3) g/d for quartile 1 and 4, respectively, with no significant difference in fish intake between the ADMA quartiles.

Patients with high ADMA levels were less likely to have been treated with PCI, having hypercholesterolemia, DM, or family history of CAD. Patients with low ADMA were more often diagnosed with ACS and significant CAD at angiography. However, the prevalence of 3-vessel disease did not differ across ADMA quartiles.

Because the majority was diagnosed with significant CAD, most patients were discharged with various medications. Antiplatelet therapy (acetylsalicylic acid and ADP receptor blockers), statins, and *β*-blockers were more frequently used by patients with low ADMA levels, whereas use of warfarin and loop diuretics was more frequent in patients with high ADMA. A total of 860 (63.0%) patients were treated with either PCI or CABG as a result of the baseline angiography.

### 3.2. Serum n-3 PUFA and Biochemical Markers according to Plasma ADMA Levels

FA and biochemical markers, relevant for CAD, by quartiles of ADMA are presented in Table 2. After adjustment for age, gender, statin therapy, and ACS, ADMA concentration was not associated with any FA (as percentage by weight (wt%) or concentration), any lipid parameter, glucose, or CRP. ADMA showed a positive association with HbA1c and creatinine and inverse association with eGFR and arginine.

### 3.3. ADMA and Risk of AMI

During the follow-up period (mean 63 (SD 20) months), a total of 129 patients experienced an AMI, of which 44 were fatal. The relationship between ADMA levels and subsequent risk of AMI after angiography was evaluated across ADMA quartiles using lower quartile 1 as reference, and for the upper decile compared to ADMA below the upper decile.

ACS and extent of CAD were strongly associated with ADMA and were included in a multivariate adjusted survival model together with other important risk factors for AMI. Hazard ratios (HR (95% CI)) for AMI according to ADMA levels are presented in Table 3. We observed only a weak, nonsignificant trend of an increased risk of AMI across ADMA quartiles. However, patients with ADMA levels in the upper decile had a significantly increased risk compared with the rest; HR 2.11 (1.34, 3.32), *P* = 0.001. Further adjustment for eGFR, hypercholesterolemia, HbA1c, lipid parameters, CRP, or coronary revascularization following baseline angiography (PCI or CABG) only minimally affected the estimate (data not shown).

### 3.4. Stratification by n-3 PUFA

**Figure 1:**
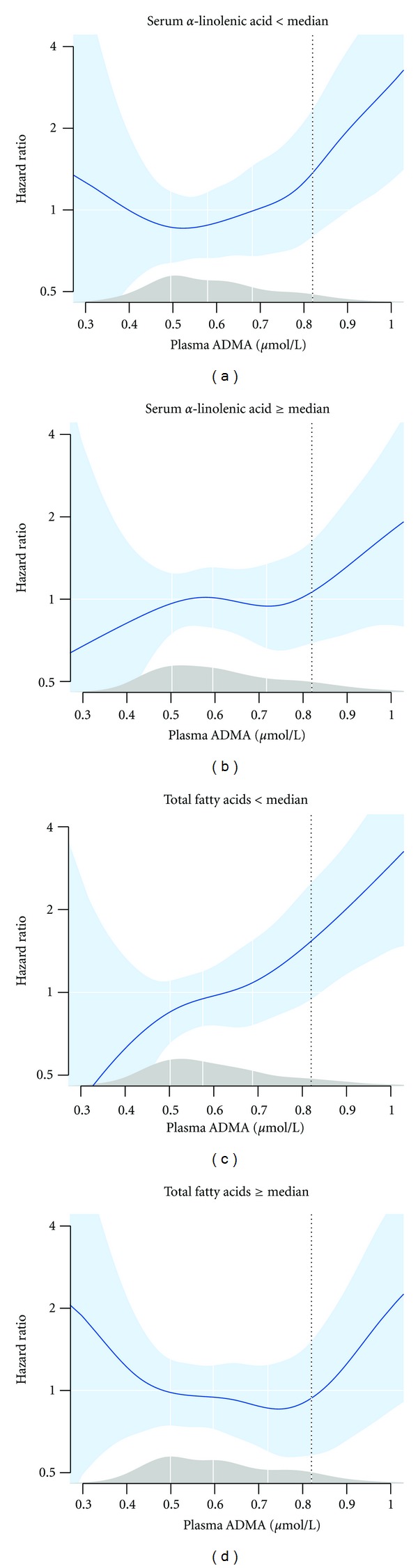
Association between plasma ADMA levels (*μ*mol/L) and acute myocardial infarction in subsets of the study population with low/high serum levels of *α*-linolenic acid (upper panels) or total fatty acids (lower panels). Median serum levels of the specified fatty acid were used for the dichotomous separation of the study subjects. The nonlinear smoothing splines estimate of the hazard ratio were estimated with additive Cox proportional hazard regression models adjusted for age (continuous), sex, diabetes mellitus (yes/no), current smoking (yes/no), acute coronary syndrome (yes/no), extend of coronary artery disease (0–3), and left ventricular ejection fraction (continuous). The solid line represents the hazard ratio, and the shaded area represents the 95% CI. The density plot on top of the *x*-axis shows the distribution of plasma ADMA in the study population and the white vertical lines denote the first quartile, median, and third quartile, respectively; the dotted vertical line marks the population 90th percentile.

Possible effect modifications of n-3 PUFA on the relationship between ADMA and risk of AMI were evaluated by repeating the survival analyses after stratifying the study population according to median levels of TFA concentration or wt% of ALA, n-3 LCPUFA (Table 4) and EPA, and DPA and DHA individually (data not shown). We observed particularly strong and significant risk associations among patients with below median levels (wt%) of ALA (HR 3.12 (1.64, 5.93)) and TFA (HR 2.60 (1.41, 4.80)) whereas no significant risk associations were observed with higher levels of ALA and TFA (Table 4, Figure 1). Effect modification was, however, close to being statistically significant only according to median ALA (*P* = 0.07) (Table 4). We observed no effect modification according to wt% of n-3 LCPUFA (Table 4) or EPA, and DPA and DHA individually (data not shown), with almost identical and statistically significant risk estimates in those with levels above and below the respective median concentrations. However, after additional adjustment for HbA1C, hypercholesterolemia, and eGFR, the risk association was strengthened in those with below median levels of n-3 LCPUFA, HR 2.81 (1.28, 6.16), *P* = 0.01, whereas the association was attenuated and no longer statistically significant in those with n-3 LCPUFA levels above median. Further adjustment for CRP or triglycerides did not appreciably alter our results.

## 4. Discussion

In this prospective cohort study, we identified plasma ADMA levels in the upper decile to be moderately associated with risk of AMI. No serum n-3 PUFA was related to plasma ADMA concentration. However, the risk of AMI associated with elevated ADMA was particularly strong among patients with ALA concentration below median, whereas similar effect modification for n-3 LCPUFA was only observed after additional adjustment.

Plasma ADMA levels in healthy individuals appears to lie in the range of 0.4–0.6 *μ*mol/L [27]. Previous studies have found elevated ADMA levels to be predictive of future AMI events. Increased ADMA levels in men with ACS have been associated with an 81% increased risk of AMI [28]. A recent population study in women reported a 75% increased risk of stroke or acute AMI in those with ADMA levels ≥ 0.71 *μ*mol/L [29]. ADMA levels which are associated with CVD or mortality vary greatly [30]. This can be due to differences in population characteristics, endpoints, sample handling and use of analytical methods. In the present study, there was a two-fold increased risk of AMI among participants with plasma ADMA levels in the upper decile (≥0.82 *μ*mol/L).

An experimental animal study demonstrated that plasma ADMA levels were reduced by EPA and DHA supplementation [16]. We therefore investigated if serum levels of different n-3 PUFA modified the association between the risk of AMI and circulating ADMA. However, we observed no association between serum n-3 PUFA levels and plasma ADMA levels, which is in agreement with a recently published intervention study from Norway showing no effect of n-3 PUFA supplementation on ADMA levels in males with long-standing hyperlipidemia [15]. Although we did not detect significant interactions, the risk of AMI related to ADMA was particularly strong among patients with levels of ALA below median, whereas no association was observed among patients with higher levels. The association between risk of AMI and ADMA was also strong in those with TFA below median. Notably, treatment with statins affects serum concentrations of some FAs [31], but adjustment for statin treatment did not alter our results, suggesting that the observed effect modifications are not induced by statin treatment. Further research is needed to clarify whether the protective effects of TFAs are due to the presence of specific FAs other than n-3 PUFA.

Previous studies have revealed a positive relation between serum glucose levels and ADMA [32]. A possible explanation may be a downregulation of DDAH-II by high levels of ROS generated by high levels of glucose [5]. We found HbA1c, which reflects glucose concentrations over a prolonged period of time, to be positively correlated to ADMA, but not to any n-3 PUFAs. When HbA1c was included in the multivariate model, the risk of AMI associated with ADMA was strengthened and significant in patients with below median levels of n-3 LCPUFA, whereas there were no associations in patients with above median levels. These data indicate that the complex interaction between glucose and n-3 PUFA metabolisms is important for the observed associations between elevated ADMA and increased risk of AMI.

A high level of total cholesterol is associated with increased production of oxLDL which has the potential to inhibit ADMA degradation [6] and upregulate ADMA synthesis [7]. Levels of n-3 PUFA or ADMA did not differ according to hypercholesterolemia, but additional adjustment for hypercholesterolemia strengthened the association between ADMA and AMI in patients with below median levels of n-3 PUFA. Additionally, the concentration of serum triglycerides was borderline significantly associated with ADMA and may thus be a potential confounder and/or effect modifier. However, including serum triglycerides in the multivariate survival model did not alter the results.

Reduced renal function is associated with elevated plasma ADMA levels. Adding eGFR to our multivariate survival model strengthened the association between ADMA and AMI in patients with below median levels of n-3 LCPUFA.

FAs of the n-3 PUFA family have anti-inflammatory properties [33] and increased levels would potentially downregulate the level of inflammatory markers, such as TNF-*α*. Including CRP in our multivariate model did not affect the association between the risk of AMI and ADMA, across levels of n-3 PUFA or TFA. Additionally, no correlation between CRP and ADMA was observed after adjustment for ACS. Based on these results, it is unlikely that the observed risk modifications are due to reduced inflammation.

This study is based on a large, well-characterized population with complete followup with respect to clinical endpoints. However, despite the clear differences in risk associations observed, even this cohort was too small to demonstrate significant effect modification. Limitations also include the single baseline measurement of FAs and biomarkers, which may have introduced underestimated associations (regression dilution bias) [34]. Furthermore, membranes of erythrocytes are less sensitive to recent FAs intake [35, 36] and would probably give a more accurate picture of the body's content of FAs or long-term FAs intake than serum levels do. The survival model was adjusted for important covariates such as DM, current smoking, ACS, hypertension, extent of CAD, and LVEF without materially altering our findings. However, residual confounding cannot be ruled out. Moderate-to-strong correlations between intake and plasma concentrations of FAs have been observed [37]. The importance of diet for the current results should therefore be determined in further studies.

## 5. Conclusions

The association between plasma ADMA and risk of AMI was influenced by serum n-3 PUFA and primarily ALA. Additional research is needed to further elucidate the clinical implications of these findings and whether the relationship between ADMA and AMI is modified by other FAs.

## Figures and Tables

**Table 1:** Baseline characteristics of participants by quartiles and in the upper decile of plasma ADMA concentration^1^.

	Quartiles					Upper decile
	1	2	3	4	
	0.46 (0.10, 0.50)	0.54 (0.50, 0.59)	0.63 (0.59, 0.70)	0.80 (0.70, 1.71)	*P* trend^2^	0.89 (0.82, 1.71)
	*n* = 342	*n* = 339	*n* = 342	*n* = 341		*n* = 136
Male sex, *n* (%)	287 (83.9)	261 (77.0)	249 (72.8)	222 (65.1)	<0.001	90 (66.2)
Age (years), mean (±SD)	58 (±9.7)	61 (±9.7)	62 (±10.7)	64 (±11.0)	<0.001	65.5 (±11.2)
BMI (kg/m^2^), mean (±SD)	27.1 (±3.55)	26.6 (±3.51)	26.4 (±4.15)	26.5 (±3.84)	0.02	26.3 (±4.08)
Fasting, *n* (%)^3^	44 (14.2)	58 (18.5)	53 (16.0)	32 (9.5)	0.05	10 (7.4)

Cardiovascular history, *n* (%)						

Previous AMI	130 (38.0)	143 (42.2)	130 (38.0)	150 (44.0)	0.20	62 (45.6)
Previous CBV	12 (3.5)	18 (5.3)	28 (8.2)	28 (8.2)	0.11	15 (11.0)
Previous PVD	30 (8.8)	25 (7.4)	34 (9.9)	47 (13.8)	0.09	23 (16.9)
Previous PCI	80 (23.4)	63 (18.6)	43 (12.6)	53 (15.5)	0.01	22 (16.2)
Previous CABG	32 (9.4)	41 (12.1)	36 (10.5)	25 (7.3)	0.05	14 (10.3)

Cardiovascular risk factors, *n* (%)						

Hypercholesterolemia^4^	212 (65.2)	199 (61.8)	189 (58.2)	155 (49.8)	<0.001	48 (40.0)
Hypertension	154 (45.0)	159 (46.9)	163 (47.7)	174 (51.0)	0.87	71 (52.2)
Impaired LVEF^5^	40 (11.7)	31 (9.1)	35 (10.2)	45 (13.2)	0.45	18 (13.2)
Diabetes^6^	41 (12.0)	38 (11.2)	29 (8.5)	32 (9.4)	0.04	12 (8.8)
Current smoker	118 (34.5)	113 (33.3)	123 (36.0)	103 (30.2)	0.22	41 (30.1)
Ex-smoker	249 (72.8)	254 (74.9)	251 (73.6)	267 (78.3)	0.86	58 (42.6)
Never smoked	74 (22.5)	94 (27.7)	78 (22.9)	101 (29.6)	0.31	37 (27.2)
Family history of CAD^7^	123 (36.4)	112 (33.3)	114 (34.0)	86 (25.8)	0.02	34 (25.4)

Clinical diagnosis before BCA, *n* (%)						

Stable angina pectoris	288 (84.2)	318 (93.8)	330 (96.5)	337 (98.8)	<0.001	135 (99.3)
Acute coronary syndrome	54 (15.8)	21 (6.2)	12 (3.5)	4 (1.2)	<0.001	1 (0.7)

Extent of CAD at BCA, *n* (%)						

No significant CAD	15 (4.4)	8 (2.4)	55 (16.1)	84 (24.6)	<0.001	33 (24.3)
1 vessel disease	112 (32.7)	118 (34.8)	83 (24.3)	55 (16.1)	<0.001	16 (11.8)
2 vessel disease	107 (31.3)	99 (29.2)	88 (25.7)	65 (19.1)	<0.001	25 (18.4)
3 vessel disease	98 (28.7)	102 (30.1)	97 (28.4)	106 (31.1)	0.29	51 (37.5)

Medication following BCA, *n* (%)						

Acetylsalicylic acid	318 (93.0)	314 (92.6)	277 (81.0)	266 (78.0)	<0.001	106 (77.9)
Statins	306 (89.5)	299 (88.2)	264 (77.2)	238 (69.8)	<0.001	83 (61.0)
*β*-blockers	270 (79.2)	271 (79.9)	241 (70.5)	240 (70.6)	0.001	89 (65.4)
ADP receptor blocker	132 (38.6)	97 (28.6)	56 (16.4)	37 (10.9)	<0.001	15 (11.0)
Anticoagulants (warfarin)	4 (1.2)	8 (2.4)	21 (6.1)	23 (6.7)	<0.001	5 (3.7)
ACE inhibitors	61 (17.8)	64 (18.9)	62 (18.1)	85 (24.9)	0.08	43 (31.6)
Angiotensin II receptor antagonist	41 (12.0)	37 (10.9)	22 (6.4)	34 (10.0)	0.06	13 (9.6)
Loop diuretics	22 (6.4)	29 (8.6)	34 (9.9)	60 (17.6)	0.001	28 (20.6)

CR following BCA, *n* (%)						

PCI	214 (62.6)	193 (56.9)	140 (40.9)	91 (26.7)	<0.001	37 (27.2)
CABG	51 (14.9)	53 (15.6)	61 (17.8)	64 (18.8)	0.34	29 (21.3)

ACE: angiotensin converting enzyme; ADP: adenosine diphosphate; BCA: baseline coronary angiography; BMI: body mass index; CABG: coronary artery bypass graft surgery; CAD: coronary artery disease; CBV: cerebrovascular disease; CR: coronary revascularization; LVEF: left ventricular ejection fraction; PCI: percutaneous coronary intervention; PVD: peripheral vascular disease.

^1^Median (range) plasma ADMA concentrations (*μ*mol/L) are presented.

^2^ *P* trend by linear (for continuous variables) and logistic (for binary variables) adjusting for age (continuous) and sex.

^3^Not having ingested any food 6 hours prior to blood samples were collected.

^4^≥6.5 mmol/L.

^5^<50%.

^6^Includes diabetes type 1 and 2.

^7^Includes those reporting to have at least one 1st degree relative suffering from CAD before the age of 55 for men and 65 for women.

**Table 2:** Serum fatty acids and biochemical markers by ADMA quartiles and in the upper decile of plasma ADMA concentration^1^.

	Quartiles					Upper decile
	1	2	3	4	*P* trend^2^
	0.46 (0.10, 0.50) *n* = 342	0.54 (0.50, 0.59) *n* = 339	0.63 (0.59, 0.70) *n* = 342	0.80 (0.70, 1.71) *n* = 341		0.89 (0.82, 1.71) *n* = 136
Fatty acids						

TFAs (mg/L)	4277 (4087, 4467)	4024 (3839, 4209)	4115 (3932, 4297)	4373 (4192, 4554)	0.73	4265 (3980, 4550)
Total n-3 PUFA (wt%)^3^	7.57 (7.23, 7.91)	7.29 (7.00, 7.59)	7.70 (7.38, 8.01)	7.82 (7.51, 8.13)	0.55	7.23 (6.74, 7.73)
ALA (wt%)	0.73 (0.71, 0.76)	0.72 (0.70, 0.74)	0.74 (0.72, 0.76)	0.75 (0.73, 0.78)	0.74	0.77 (0.73, 0.80)
n-3 LCPUFA (wt%)^4^	6.90 (6.57, 7.23)	6.52 (6.19, 6.84)	6.86 (6.54, 7.18)	6.84 (6.52, 7.15)	0.54	6.47 (5.97, 6.97)

Lipid related parameters						

ApoA1 (g/L)	1.36 (1.33, 1.39)	1.35 (1.32, 1.38)	1.36 (1.33, 1.39)	1.36 (1.34, 1.39)	0.51	1.33 (1.29, 1.37)
ApoB (g/L)	0.94 (0.91, 0.97)	0.91 (0.88, 0.94)	0.95 (0.92, 0.97)	0.95 (0.92, 0.98)	0.59	0.93 (0.89, 0.97)
Total Ch. (mmol/L)	5.26 (5.13, 5.40)	5.10 (4.96, 5.23)	5.29 (5.16, 5.42)	5.31 (5.17, 5.44)	0.58	5.17 (4.96, 5.38)
LDL Ch. (mmol/L)	3.19 (3.07, 3.30)	3.14 (3.02, 3.25)	3.32 (3.20, 3.43)	3.30 (3.19, 3.41)	0.71	3.23 (3.05, 3.40)
HDL Ch. (mmol/L)	1.30 (1.26, 1.34)	1.28 (1.24, 1.32)	1.32 (1.28, 1.36)	1.33 (1.29, 1.37)	0.41	1.28 (1.21, 1.34)
Non HDL (mmol/L)	1.30 (1.26, 1.34)	1.28 (1.24, 1.32)	1.32 (1.28, 1.36)	1.33 (1.29, 1.37)	0.42	3.89 (3.68, 4.10)
TG (mmol/L)	1.96 (1.80, 2.12)	1.73 (1.58, 1.89)	1.66 (1.51, 1.81)	1.77 (1.62, 1.92)	0.06	1.72 (1.49, 1.96)
Lp(a) (mmol/L)	0.37 (0.33, 0.41)	0.37 (0.33, 0.41)	0.37 (0.33, 0.41)	0.39 (0.35, 0.43)	0.23	0.40 (0.39, 0.47)

Other parameters						

Glucose (mmol/L)	6.46 (6.18, 6.74)	6.27 (6.00, 6.55)	6.23 (5.96, 6.49)	6.22 (5.95, 6.48)	0.15	6.28 (5.87, 6.70)
HbA1c (mmol/L)	5.97 (5.82, 6.12)	5.79 (5.64, 5.93)	5.91 (5.77, 6.05)	6.40 (6.25, 6.54)	<0.001	6.56 (6.33, 6.78)
Arginine (*μ*mol/L)	76.5 (73.8, 79.2)	80.9 (78.3, 83.5)	70.6 (68.0, 73.1)	53.6 (51.1, 56.2)	<0.001	49.9 (45.7, 54.0)
Creatinine (*μ*mol/L)	85.4 (83.8, 89.4)	86.6 (83.8, 89.4)	88.3 (85.6, 91.0)	95.2 (92.5, 97.5)	<0.001	104.0 (99.8, 108.2)
GFR (mL/min)	90.3 (88.8, 91.7)	88.7 (87.2, 90.1)	87.1 (85.6, 88.5)	82.6 (81.2, 84.0)	<0.001	78.0 (75.8, 80.3)
CRP (mg/L)	6.33 (5.19, 7.48)	4.10 (2.98, 5.22)	3.75 (2.65, 4.85)	4.09 (3.00, 5.19)	0.99	3.72 (2.00, 5.44)

ALA: *α*-linolenic acid; ApoA1: apolipoprotein A-I; Ch: cholesterol; CRP: C-reactive protein; DHA: docosahexaenoic acid; DPA: docosapentaenoic acid; EPA: eicosapentaenoic acid; GFR: glomerular filtration rate; HbA1c: hemoglobin A1c; HDL: high density lipoprotein; LDL: low density lipoprotein; Lp(a): lipoprotein(a); n-3 PUFAs: omega-3 polyunsaturated fatty acids; TFAs: total fatty acids; TG: triglycerides; n-3 LCPUFAs: long chain omega-3 polyunsaturated fatty acids; wt%: percentage by weight.

^1^Median (range) plasma ADMA concentrations (*μ*mol/L) are presented. For fatty acids, lipid related parameters, and other parameters; mean (95% confidence interval) values are given after adjustment for age (continuous) and sex.

^2^ *P* trend by linear regression adjusting for age (continuous), sex, acute coronary syndrome (yes/no), and statin treatment at baseline (yes/no).

^3^Combination of ALA, EPA, DPA, and DHA.

^4^Combination of EPA, DPA, and DHA.

**Table 3:** Risk of acute myocardial infarction across quartiles and upper decile of ADMA.

Model	Quartiles							Upper decile		
		2		3		4	*P* trend			
	HR	95% CI	HR	95% CI	HR	95% CI		HR	95% CI	*P* value
Univariate	1.26	(0.75, 2.12)	1.23	(0.73, 2.07)	1.47	(0.89, 2.42)	0.16	2.24	(1.45, 3.47)	<0.001
Sex, age adjusted	1.22	(0.72, 2.07)	1.16	(0.69, 1.96)	1.35	(0.80, 2.25)	0.32	2.06	(1.33, 3.21)	0.001
Multivariate adjusted^1^	1.22	(0.72, 2.07)	1.27	(0.74, 2.18)	1.44	(0.84, 2.47)	0.20	2.11	(1.34, 3.32)	0.001

HR: hazard ratio; CI: confidence interval.

Hazard ratios for the quartile groups are compared to first quartile; hazard ratio for plasma ADMA levels > 90th percentile is compared to plasma ADMA levels < 90th percentile.

^1^The model includes age (continuous), sex, acute coronary syndrome (yes/no), diabetes mellitus (yes/no), hypertension (yes/no), current smoking (yes/no), extend of coronary artery disease (0–3), and left ventricular ejection fraction (continuous).

**Table 4:** Risk of acute myocardial infarction for the upper decile of ADMA in strata of TFAs and n-3 PUFA.

Fatty acids	Below median	Above median	*P* int.^1^
	HR (95% CI)	HR (95% CI)	
TFAs (mg/L)			
Model 1^2^	2.60 (1.41, 4.80)	1.67 (0.83, 3.36)	0.29
Model 2^3^	2.57 (1.25, 5.29)	1.49 (0.56, 3.93)	0.35
Total n-3 PUFA (wt%)^4^			
Model 1	1.89 (0.98, 3.63)	2.25 (1.17, 4.34)	0.72
Model 2	2.36 (1.05, 5.33)	1.97 (0.91, 4.30)	0.99
ALA (wt%)			
Model 1	3.12 (1.64, 5.93)	1.49 (0.77, 2.88)	0.07
Model 2	2.42 (1.13, 5.16)	1.57 (0.69, 3.55)	0.11
n-3 LCPUFA (wt%)^5^			
Model 1	2.05 (1.08, 3.89)	2.11 (1.08, 4.15)	0.96
Model 2	2.81 (1.28, 6.16)	1.74 (0.78, 3.90)	0.78

ALA: *α*-linolenic acid; CI: confidence interval; DHA: docosahexaenoic acid; DPA: docosapentaenoic acid; EPA: eicosapentaenoic acid; HR: hazard ratio; n-3 PUFAs: omega-3 polyunsaturated fatty acids; TFAs: total fatty acids; n-3 LCPUFAs: long chain omega-3 polyunsaturated fatty acids; wt%: percentage by weight.

^1^ *P* interaction.

^2^Model 1: hazard ratios of acute myocardial infarction for plasma ADMA > 90th percentile with plasma ADMA levels < 90th percentile as reference. The model included age (continuous), sex, acute coronary syndrome (yes/no), diabetes mellitus (yes/no), hypertension (yes/no), current smoking (yes/no), extend of coronary artery disease (0–3), left ventricular ejection fraction (continuous).

^3^Model 2: hazard ratios of acute myocardial infarction for plasma ADMA levels > 90th percentile with plasma ADMA levels < 90th percentile as reference. The model included age (continuous), sex, acute coronary syndrome (yes/no), diabetes mellitus (yes/no), hypertension (yes/no), current smoking (yes/no), extend of coronary artery disease (0–3), left ventricular ejection fraction (continuous), hypercholesterolemia (yes/no), HbA1c (continuous), and glomerular filtration rate (continuous).

^4^Combination of ALA, EPA, DPA, and DHA.

^5^Combination of EPA, DPA, and DHA.

## References

[1] Kapadia MR, Chow LW, Tsihlis ND (2008). Nitric oxide and nanotechnology: a novel approach to inhibit neointimal hyperplasia. *Journal of Vascular Surgery*.

[2] Dulak J, Jozkowicz A, Dembinska-Kiec A (2000). Nitric oxide induces the synthesis of vascular endothelial growth factor by rat vascular smooth muscle cells. *Arteriosclerosis, Thrombosis, and Vascular Biology*.

[3] Cooke JP (2003). Flow, NO, and atherogenesis. *Proceedings of the National Academy of Sciences of the United States of America*.

[4] Vallance P, Leone A, Calver A, Collier J, Moncada S (1992). Accumulation of an endogenous inhibitor of nitric oxide synthesis in chronic renal failure. *The Lancet*.

[5] Sorrenti V, Mazza F, Campisi A, Vanella L, Li Volti G, di Giacomo C (2006). High glucose-mediated imbalance of nitric oxide synthase and dimethylarginine dimethylaminohydrolase expression in endothelial cells. *Current Neurovascular Research*.

[6] Ito A, Tsao PS, Adimoolam S, Kimoto M, Ogawa T, Cooke JP (1999). Novel mechanism for endothelial dysfunction: dysregulation of dimethylarginine dimethylaminohydrolase. *Circulation*.

[7] Böger RH, Sydow K, Borlak J (2000). LDL cholesterol upregulates synthesis of asymmetrical dimethylarginine in human endothelial cells: involvement of S-adenosylmethionine-dependent methyltransferases. *Circulation Research*.

[8] Wakino S, Hayashi K, Tatematsu S (2005). Pioglitazone lowers systemic asymmetric dimethylarginine by inducing dimethylarginine dimethylaminohydrolase in rats. *Hypertension Research*.

[9] Ivashchenko CY, Bradley BT, Ao Z, Leiper J, Vallance P, Johns DG (2010). Regulation of the ADMA-DDAH system in endothelial cells: a novel mechanism for the sterol response element binding proteins, SREBP1c and -2. *American Journal of Physiology*.

[10] Xu HE, Lambert MH, Montana VG (1999). Molecular recognition of fatty acids by peroxisome proliferator-activated receptors. *Molecular Cell*.

[11] Xu J, Teran-Garcia M, Park JHY, Nakamura MT, Clarke SD (2001). Polyunsaturated fatty acids suppress hepatic sterol regulatory element-binding protein-1 expression by accelerating transcript decay. *Journal of Biological Chemistry*.

[12] Mozaffarian D (2005). Does alpha-linolenic acid intake reduce the risk of coronary heart disease? A review of the evidence. *Alternative Therapies in Health and Medicine*.

[13] Kwak SM, Myung SK, Lee YJ, Seo HG, Korean Meta-analysis Study Group (2012). Efficacy of omega-3 fatty acid supplements (eicosapentaenoic acid and docosahexaenoic acid) in the secondary prevention of cardiovascular disease: a meta-analysis of randomized, double-blind, placebo-controlled trials. *Archives of Internal Medicine*.

[14] Harper CR, Jacobson TA (2005). Usefulness of omega-3 fatty acids and the prevention of coronary heart disease. *American Journal of Cardiology*.

[15] Eid HMA, Arnesen H, Hjerkinn EM, Lyberg T, Ellingsen I, Seljeflot I (2006). Effect of diet and omega-3 fatty acid intervention on asymmetric dimethylarginine. *Nutrition and Metabolism*.

[16] Raimondi L, Lodovici M, Visioli F (2005). n-3 polyunsaturated fatty acids supplementation decreases asymmetric dimethyl arginine and arachidonate accumulation in aging spontaneously hypertensive rats. *European Journal of Nutrition*.

[17] Fard A, Tuck CH, Donis JA (2000). Acute elevations of plasma asymmetric dimethylarginine and impaired endothelial function in response to a high-fat meal in patients with type 2 diabetes. *Arteriosclerosis, Thrombosis, and Vascular Biology*.

[18] Ebbing M, Bleie Ø, Ueland PM (2008). Mortality and cardiovascular events in patients treated with homocysteine-lowering B vitamins after coronary angiography: a randomized controlled trial. *Journal of the American Medical Association*.

[19] Manger MS, Strand E, Ebbing M (2010). Dietary intake of n-3 long-chain polyunsaturated fatty acids and coronary events in Norwegian patients with coronary artery disease. *American Journal of Clinical Nutrition*.

[20] (2002). Biochemical verification of tobacco use and cessation. *Nicotine and Tobacco Research*.

[21] Alpert JS, Thygesen K, Antman E, Bassand JP (2000). Myocardial infarction redefined—a consensus document of The Joint European Society of Cardiology/American College of Cardiology Committee for the redefinition of myocardial infarction. *European Heart Journal*.

[22] Kates M, Dates M (1986). *General Analytical ProcEdures. Techniques of Lipidology*.

[23] Grimstad T, Bjørndal B, Cacabelos D (2012). Dietary supplementation of krill oil attenuates inflammation and oxidative stress in experimental ulcerative colitis in rats. *Scandinavian Journal of Gastroenterology*.

[24] Midttun O, Hustad S, Ueland PM (2009). Quantitative profiling of biomarkers related to B-vitamin status, tryptophan metabolism and inflammation in human plasma by liquid chromatography/tandem mass spectrometry. *Rapid Communications in Mass Spectrometry*.

[25] Levey AS, Stevens LA, Schmid CH (2009). A new equation to estimate glomerular filtration rate. *Annals of Internal Medicine*.

[26] Therneau TM, Grambsch PM (2000). *Modeling Survival Data—Extending the Cox Model*.

[27] Horowitz JD, Heresztyn T (2007). An overview of plasma concentrations of asymmetric dimethylarginine (ADMA) in health and disease and in clinical studies: methodological considerations. *Journal of Chromatography B*.

[28] Cavusoglu E, Ruwende C, Chopra V (2009). Relationship of baseline plasma ADMA levels to cardiovascular outcomes at 2 years in men with acute coronary syndrome referred for coronary angiography. *Coronary Artery Disease*.

[29] Leong T, Zylberstein D, Graham I (2008). Asymmetric dimethylarginine independently predicts fatal and nonfatal myocardial infarction and stroke in women: 24-year follow-up of the population study of women in Gothenburg. *Arteriosclerosis, Thrombosis, and Vascular Biology*.

[30] Böger RH, Maas R, Schulze F, Schwedhelm E (2009). Asymmetric dimethylarginine (ADMA) as a prospective marker of cardiovascular disease and mortality—an update on patient populations with a wide range of cardiovascular risk. *Pharmacological Research*.

[31] Harris JI, Hibbeln JR, Mackey RH, Muldoon MF (2004). Statin treatment alters serum n-3 and n-6 fatty acids in hypercholesterolemic patients. *Prostaglandins Leukotrienes and Essential Fatty Acids*.

[32] Abbasi F, Asagmi T, Cooke JP (2001). Plasma concentrations of asymmetric dimethylarginine are increased in patients with type 2 diabetes mellitus. *American Journal of Cardiology*.

[33] Wall R, Ross RP, Fitzgerald GF, Stanton C (2010). Fatty acids from fish: the anti-inflammatory potential of long-chain omega-3 fatty acids. *Nutrition Reviews*.

[34] Hu FB, Stampfer MJ, Rimm E (1999). Dietary fat and coronary heart disease: a comparison of approaches for adjusting for total energy intake and modeling repeated dietary measurements. *American Journal of Epidemiology*.

[35] Sun Q, Ma J, Campos H, Hankinson SE, Hu FB (2007). Comparison between plasma and erythrocyte fatty acid content as biomarkers of fatty acid intake in US women. *American Journal of Clinical Nutrition*.

[36] Katan MB, Deslypere JP, Van Birgelen APJM, Penders M, Zegwaard M (1997). Kinetics of the incorporation of dietary fatty acids into serum cholesteryl esters, erythrocyte membranes, and adipose tissue: an 18-month controlled study. *Journal of Lipid Research*.

[37] Willett W (1998). *Nutritional Epidemiology*.

